# Institutional, neighborhood, and life stressors on loneliness among older adults

**DOI:** 10.1186/s12889-025-21463-7

**Published:** 2025-01-29

**Authors:** Kazumi Tsuchiya, Amy Danielle Thierry, Harry Owen Taylor

**Affiliations:** 1 Dalla Lana School of Public Health, University of Toronto, 155 College St, Toronto, ON M5T 3M7 Canada; 2 Department of Public Health Sciences, Xavier University of Louisiana, 1 Drexel Drive, New Orelans, LA 70125 USA; 3 Faculty of Social Work, University of Toronto, 246 Bloor St W, Toronto, ON M5S 1V4 Canada

**Keywords:** Discrimination; Neighborhood characteristics; stress, Loneliness, Health disparities, Health and Retirement Study

## Abstract

**Background:**

Loneliness is a public health epidemic in the United States (US), with older adults being vulnerable to experiencing loneliness. Predictors of loneliness are less understood among racial/ethnic groups of US older adults, and few studies have included perceived institutional discrimination (PID), stressful life events (SLE), and perceived neighborhood characteristics (PNC) as antecedent stressors of loneliness in diverse older adult samples. Our study assessed the relationship between these stressors and loneliness among specific racial/ethnic groups of older adults.

**Methods:**

We used the Health and Retirement Study data (*n* = 9,904) to examine whether PID, SLE, and PNC were associated with loneliness. Loneliness was measured using the 11-item UCLA Loneliness Scale. PID included unfairly not hired for a job, unfairly prevented from moving into a neighborhood, and unfairly treated by the police. SLE included moving to a worse neighborhood/residence, being robbed or burglarized, and unemployed/looking for a job. PNC were measured as discohesion and disorder. Lagged multivariate linear regression models regressed loneliness (2014/2016 HRS waves) on PID, SLE and PNC (2010/2012 HRS waves) measured as cumulative totals and individual items. Models were stratified by Black (BOAs), Hispanic/Latinx (HOAs), and White (WOAs) older adults.

**Results:**

Cumulative totals of PID, SLE, and neighborhood discohesion were associated with loneliness among BOAs while only discohesion was associated with loneliness among HOAs. Cumulative totals for PID, SLE, and PNC were associated with loneliness among WOAs. Individual stressors predicting loneliness for BOAs were moving to a worse residence and being robbed/burglarized. For HOAs, being prevented from moving to a neighborhood was associated with greater loneliness while being robbed/burglarized was associated with less loneliness. Individual stressors predicting greater loneliness for WOAs were being unfairly not hired for a job, receiving unfair treatment during police encounters, and moving to a worse residence.

**Conclusions:**

Our study finds racial/ethnic variation in psychosocial stressors predicting loneliness four years later. Nevertheless, neighborhood discohesion was the most salient stressor and was associated with greater loneliness across all racial/ethnic groups. Future research and interventions should consider the differing stress appraisal processes across groups and to support the development of resources and policies to ameliorate loneliness among diverse older adults.

**Supplementary Information:**

The online version contains supplementary material available at 10.1186/s12889-025-21463-7.

## Background

Loneliness has become increasingly recognized as a public health issue, especially among older adults. It is defined as when an individual perceives their social relationships to be deficient in quantity and/or quality, or feels alone [1]. Loneliness has been associated with poor health outcomes among older adults, including depression, functional limitations, and cardiovascular disease [2, 3]. Additionally, loneliness has been associated with worse cognitive health outcomes and increased mortality risk in older adult populations [4–6]. Numerous reports, including from the United States (US) Surgeon General, have emphasized the importance of social connections as foundational to one’s survival and developing and maintaining one’s health [7, 8]. Hence, loneliness is considered to be a critical public health issue in the US and globally, with older adults being a population particularly vulnerable to adverse health outcomes related to loneliness. Given the increasing population of older adults in the US and other countries [9], loneliness is an important factor to address to support healthy aging for community-dwelling older adults.

A growing body of research has focused on understanding predictors of loneliness in later life. Loneliness may be differentially distributed among older adults, and processes leading to loneliness may vary across different groups of older adults, which can have important implications in later life. Recent research among adults in the US found that loneliness is highest at middle age and among adults > 80 years of age [10]. Generally, older adults have an increased risk for experiencing loneliness due to impaired health, smaller social networks, and limited opportunities to form new social connections in their communities [5, 11]. Furthermore, research has documented that people with chronic health conditions and poor self-rated health are more likely to feel lonely than their healthier counterparts [12–15]. 

Nonetheless, less is known about perceived institutional discrimination, stressful life events, and perceived neighborhood characteristics as antecedents of loneliness, particularly among diverse aging populations. Because racially/ethnically minoritized groups with long-standing documented health disparities make up a growing segment of the US older adult population [9], understanding critical drivers of loneliness can help to elucidate possible points of intervention towards improving health equity. Scholars have begun to examine risk factors for experiencing loneliness and its related negative health outcomes among marginalized populations. Some research has documented that loneliness is similar or less prevalent among Black older adults compared to White older adults [15, 16], while other studies have found loneliness to be greater in Black and Hispanic/Latinx older adults [17, 18]. These differences in findings may be due to the fact that residing in stressful, low-resourced neighborhoods, which are disproportionately composed of Black and Hispanic/Latinx individuals, may promote loneliness through various psychosocial pathways. Still, there remains a gap in the literature in our understanding of which institutional, community, and contextual factors may be more strongly tied to loneliness, particularly in understanding the role of stressors stemming from institutional and neighborhood-level risk factors which may vary across racial/ethnic groups in the experience of loneliness [19]. 

## Racism, residential segregation, & loneliness

Robust evidence has found that communities of color, including Black and Hispanic/Latinx communities, experience a greater burden of adverse health outcomes [20, 21]. These health inequities are due to the ubiquitous nature of structural racism and its deeply entrenched systems that privilege White individuals over communities of color [22–24]. Structural racism is defined as the total sum of the ways in which society promotes racism and reinforces the unjust distribution of resources through multiple interconnected systems [23]. Additionally, racism is enacted through numerous institutions, including employment, housing, and policing, that are intrinsically connected in creating deleterious neighborhood conditions in which racially/ethnically minoritized groups are more likely to live in than White adults [25]. 

Previous research has shown that as a byproduct of structural racism, racial residential segregation contributes to limited access to quality education, constrained employment opportunities, higher levels of policing, and disinvestment of neighborhood infrastructure and resources for racially/ethnically minoritized communities in comparison to White communities [26]. Black and Hispanic/Latinx people are more likely to reside in adverse social and physical conditions, including greater environmental hazards and limited economic and political resources that often characterize racially segregated neighborhoods [24, 26–28], which in turn, impact health [21, 29]. For example, structural racism enacted at the neighborhood level, such as redlining and housing discrimination, has been associated with worse self-rated health and greater number of chronic conditions among racially/ethnically minoritized adults [30, 31]. Lack of access to affordable and safe neighborhoods can negatively affect the health of older adults, particularly racially/ethnically minoritized adults, via exposure to harmful built environmental factors, increased stress, limited availability of psychosocial supports, and housing-related financial strain that may impede upon access to healthcare and other health-promoting resources [31, 32]. However, the relationship between structural racism measured at the neighborhood level and loneliness, an important public health indicator, remains an under-examined mechanism that may link structural racism to health.

The resultant effects of racism are experienced at the neighborhood level via discrimination [33, 34], which has implications for relationships (or relationship strain) and in turn, loneliness among Black and Hispanic/Latinx adults. Several studies have found that discrimination impacts the quality of close relationships, including spouses, among Black and Hispanic/Latinx adults [35–39]. A recent study using a sample of midlife and older adults in the Health and Retirement Study found that perceived everyday discrimination was associated with greater loneliness, even after controlling for sociodemographic factors, levels of social contact, and social support [40]. Previous research has also found that experiencing discrimination is associated with neighborhood social capital [41]. In other words, perceived discrimination erodes the development and maintenance of strong social relationships among neighborhood residents. These findings suggest that perceived discrimination may contribute to loneliness due to challenges in establishing positive relationships among community-dwelling older adults. However, this area of research linking perceived discrimination and loneliness is underdeveloped. Specifically, if and how experiences of perceived institutional discrimination (i.e., housing, policing, and employment), along with other neighborhood-level conditions (i.e., social discohesion and physical disorder), may be associated with loneliness among diverse older adults remains unclear.

### Neighborhood context & loneliness

Emerging research has begun to examine how neighborhood conditions contribute to loneliness. The neighborhood context may be particularly important for older adults who tend to spend more time daily within their neighborhoods than younger people as they are less likely to leave their home/residence, [42] in tandem with older adults in the US striving to age in place [43]. Older adults may have a greater reliance on their neighbors due to limited mobility [44], decreased social network size, and limited contact with others [45]. Thus, loneliness among older adults may be attributed to aspects of the neighborhood that inhibit social interactions and reduce feelings of belonging in one’s community.

Objective and perceived neighborhood characteristics capture different aspects of how individuals experience their neighborhoods and each may contribute to health via different mechanisms, such as through behaviors, psychological stress, and social relationships [46]. Recent research demonstrates that greater availability and accessibility of services in the neighborhood has been linked to lower levels of loneliness [47–50], while higher rates of crime have been associated with more loneliness [51]. Some research demonstrates perceived neighborhood characteristics are linked to both physical and mental health among adults [46, 52, 53], with other research indicating that subjective neighborhood measures were more strongly tied to self-rated health than objective neighborhood factors [54]. Given these findings, perceived neighborhood characteristics are also important to consider in regards to loneliness among older adults. Negative neighborhood perceptions influenced by objective neighborhood conditions (e.g., objective physical disorder) may prime individuals to have a more negative or threatening perception of their environment, including of others living in their neighborhood, thus contributing to greater loneliness [55, 56]. Higher levels of perceived neighborhood disorder, particularly fear of crime or greater vandalism, among older adults may hinder their likelihood of spending time outdoors and engaging in social interactions with their neighbors, which can adversely affect their mental and physical health [16, 57]. Low perceived neighborhood social cohesion, which includes older adults’ ability to build social connections with neighbors and receive social support [58], may increase older adults’ feelings of being disconnected from their neighbors and subsequently lead to increased loneliness [47, 48, 50, 59]. Moreover, Black and Hispanic/Latinx older adults are more likely to hold negative perceptions of their neighborhoods, specifically physical disorder and social cohesion, compared to White older adults [60, 61]. Given their increased exposure to structural racism through interconnecting institutions which influence local communities, Black and Hispanic/Latinx older adults may experience heightened vulnerability to adverse neighborhood conditions [11, 22], and in turn, greater risk for loneliness.

### Stressors & loneliness

Related to discrimination and neighborhood conditions, life stressors may be implicated in the experience of loneliness, whereby greater stress may increase feelings of loneliness as well as exacerbate the detrimental health effects of loneliness. High levels of stress may contribute to individuals having more negative perceptions of their current social-environmental contexts and withdrawing from their social networks. In elucidating pathways of loneliness, previous studies have explored how differential levels of stress exposures may impact loneliness [62]. Chronic stress has been associated with increased loneliness [13, 14, 18], and more frequent experiences of everyday stressors is also associated with increasing levels of loneliness [63]. Other scholars have elucidated that significant stressful life events and life transitions, or triggers, can contribute to the onset and development of loneliness [64]. These triggers may include retirement, moving, divorce, illness, death of a spouse, friend, or family members, and other stressors (e.g., discrimination, financial strain) [11]. Multiple triggers may also co-occur during a significant life transition (e.g., moving to a new residence after the death of a spouse/partner) [11, 65].

Furthermore, racially/ethnically minoritized adults generally experience more chronic stressors than their White counterparts, whereby these groups contend with multiple stressors stemming from racism and ageism, which contribute to heightened vulnerability to the onset of loneliness. For example, major life stressors related to institutional racism and deleterious neighborhood environments, such as having to move to a worse neighborhood or home, being robbed or burglarized, and becoming unemployed may be disproportionately experienced by Black and Hispanic/Latinx individuals, and thus, increase their likelihood of being lonely and potentially exacerbate adverse health outcomes [65]. 

### Limitations of the previous research & proposed study

Several recent reviews have elucidated the role of contextual factors (i.e., neighborhood, stressors) for loneliness among older adults [47, 66]. However, few studies have investigated how multiple domains of stressors stemming from complex and nuanced factors (e.g., racism, life stressors, neighborhood characteristics) may impact loneliness among older adults by specific racial/ethnic groups [67].

The aim of the current study was to investigate whether multiple institutional discrimination events, stressful life events, and perceived neighborhood characteristics (e.g., discohesion, disorder) were associated with loneliness among White, Black, and Hispanic/Latinx adults over the age of 50 living in the US. Given the multitude of stressors experienced by racially/ethnically minoritized older adults, we also examine whether these relationships are stratified by race/ethnicity. These findings will illuminate our understanding of which types of institutional discrimination, stressful life events, and neighborhood characteristics are most salient for specific racial/ethnic groups and their experiences of loneliness.

## Methods

### Sample

Data for the current study are from the Health and Retirement Study (HRS). The HRS is one of the largest nationally representative longitudinal surveys of adults aged 50 and older living in the United States [68]. Data collection for the HRS began in 1992, with the data being collected once every two years. In 2006, the HRS began the self-administered psychosocial leave-behind questionnaire (LBQ) [68, 69]. A random half-sample of the HRS core survey participants was selected to complete the HRS LBQ in 2006, with the other half-sample completing the HRS LBQ in 2008. The HRS LBQ utilizes a rotational study design where data is collected once every four years for the same half-sample. For more information regarding the HRS LBQ, please see Smith and colleagues [69]. For the current study, we employed a lagged design where loneliness as the outcome measure (2014/2016 waves) was regressed onto predictor measures (2010/2012 waves). The total analytic sample for our study consists of 9,904 respondents. Analyses were stratified by race/ethnicity where separate models were estimated among Black, Hispanic/Latinx, and White respondents.

### Key variables

*Loneliness* is measured using the UCLA 11-item loneliness scale, a shortened version of the 20-item UCLA Loneliness Scale [69, 70]. Items from this scale include how often respondents feel “they lack companionship,” “left out,” “isolated from others,” “alone,” and, “that there are people you feel close to.” Response items are averaged together to create a mean score. Higher scores on this scale are indicative of greater loneliness. Loneliness scale scores are set to missing if more than 5 items are missing [69]. The Cronbach’s Alpha for this scale is 0.88 for the overall sample, and 0.87, 0.85, and 0.89 for Black, Hispanic/Latinx, and White older adults, respectively, indicating good reliability across each of the subsamples.

Perceived institutional discrimination and stressful life events span domains of employment, policing, and the neighborhood context/residence. *Perceived institutional discrimination* measures included whether respondents were unfairly not hired for a job, unfairly prevented from moving into a neighborhood, and unfairly stopped, searched, questioned, physically threatened and/or abused by the police. These items were adapted from previous research on perceived institutional racism [25, 71–73]. Furthermore, these variables were summed together to create a count variable of total perceived institutional discrimination as done in previous research [29]. *Stressful life events* included respondents’ report of whether they experienced the following in the past 5 years (yes/no): moved to a worse neighborhood or residence, were robbed or had their home burglarized, and were unemployed and looking for a job for the past 3 months. Each of these measures were summed together to create a count variable of total stressful life events.

Perceived neighborhood characteristics consisted of four measures: neighborhood discohesion, neighborhood disorder, neighborhood safety, and neighborhood cleanliness. Each variable is constructed from HRS participants’ responses to a set of survey items on a Likert scale from 1 (highly agree) to 7 (highly disagree). Scores for each survey question corresponding to the four neighborhood variables were summed and averaged, with higher scores indicating worse perceptions of the neighborhood characteristic. We employ similar procedures as previous empirical studies using the HRS data [60, 61, 69]. Specifically, *perceived neighborhood discohesion* was measured using responses to the following survey items: (1) feel a part of this area, (2) most people can be trusted, (3) most people are friendly, and (4) people help you if in trouble. Higher scores represent greater perceived neighborhood discohesion. *Perceived neighborhood safety* was assessed through two items about the neighborhood: (1) feel safe to walk alone after dark and (2) no problems with vandalism and graffiti. *Perceived neighborhood cleanliness* was measured using two items: (1) this area is kept very clean and (2) there are no vacant/deserted houses or storefronts. Both perceived neighborhood safety and cleanliness were reverse coded so that higher scores represent greater unsafety and uncleanliness, respectively (we refer to these measures as unsafety and uncleanliness throughout the remainder of the manuscript). *Perceived neighborhood disorder* is a combined measure that averages the four safety and cleanliness items, with higher scores representing greater perceived neighborhood disorder.

### Covariables

We included socio-demographic measures that have been included in previous studies of loneliness [74] and neighborhood conditions [60, 61]. Race/ethnicity was measured with the following categories: Black, Hispanic/Latinx, and White. US region was measured with the following categories: South, Northeast, Midwest, and West. The following variables were measured dichotomously: urbanicity (using HRS-provided 2013 Beale rural-urban continuum codes measure where urban = counties in metro areas of 1 million population or more and non-urban = counties of < 1 million population), gender (men, women), coupled status (not coupled, coupled), employment status (working, not working), and HRS LBQ baseline wave (2010, 2012). Age (range 50–96) and years of education (range 0–17) were measured continuously. Household income (range: 0 to 6.45) was measured continuously using the HRS-provided imputed variable and was log-transformed due to its skewed distribution.

### Analytic strategy

Survey weights provided by the HRS were applied to all analyses to account for the complex sampling design of the survey to obtain national estimates. Data were analyzed using STATA 18.0. Descriptive statistics were stratified by race/ethnicity. We then conducted lagged multivariable linear regression models in which loneliness measured in the 2014 and 2016 HRS waves and was regressed on explanatory/independent variables from the 2010 to 2012 HRS waves. All regression models were stratified by race/ethnicity. For our multivariable ordinary least squares (OLS) regression analyses, we conducted two sets of regression models for each racial/ethnic group for a total of 6 models. The first set of models regressed loneliness on total number of perceived institutional discrimination experiences, total number of stressful life events, neighborhood discohesion, and neighborhood disorder, adjusting for all other covariables. The second set of models tested associations between loneliness and each of the individual items for perceived institutional discrimination and stressful life events, perceived neighborhood discohesion, and the perceived neighborhood disorder measures of neighborhood unsafety and uncleanliness.

Due to the missing data across the HRS LBQ waves, data were imputed. The variables with the highest missing data were from the perceived institutional discrimination measures, which included unfairly not hired for a job (*N* = 218; 1.87%), unfairly prevented from moving into a neighborhood (*N* = 181;1.57%), and unfairly stopped, threatened, or abused by the police (*N* = 185; 1.57%). Furthermore, the other measures in our study had 1–2% missing data, including for stressful life events, perceived neighborhood factors and loneliness. As recommended by White and colleagues [75], we imputed twenty different datasets. The multivariable linear regression models are estimated across each of the twenty different datasets. Estimates derived from each dataset provide unique model parameter estimates (regression coefficients and standard errors). These parameter estimates across each dataset are combined to determine statistical significance.

## Results

### Descriptive statistics

Table 1 presents the descriptive statistics of the measures in the study. The mean score of loneliness for the entire sample was 1.53 (SD = 0.45). For total perceived institutional discrimination, the average score was 0.18 (SD = 0.46) and the average score for total stressful life events was 0.18 (SD = 0.44). In examining the perceived institutional discrimination events separately, 9.33% of the sample reported perceived discrimination in job hiring, 1.82% reported perceived discrimination in being prevented from moving to a new residence, and 6.89% reported perceived discrimination through police encounters. For each of the life stressors, we find approximately 2.74% reported moving into a worse neighborhood/residence, 5.07% reported being robbed/burglarized, and 10.66% reported being unemployed and looking for work. Average scores for perceived neighborhood characteristics were as follows: 1.51 (SD = 1.32) for neighborhood discohesion, 1.45 (SD = 1.35) for neighborhood disorder, 1.47 (SD = 1.54) for neighborhood unsafety, and 1.44 (SD = 1.42) for neighborhood uncleanliness. The mean of log-transformed income was 4.66 (SD = 0.62) and the average years of education was 13.36 (SD = 2.81). Most respondents were women, lived in non-urban areas, lived in the South, and were coupled or in a romantic relationship. A correlation analysis was also conducted among each of the continuous variables in the current study. All variables were significantly correlated at the 0.05 level (Supplemental Table 1).

**Table 1:** Univariate and bivariate statistics and frequencies for loneliness, perceived institutional discrimination, stressful life events, neighborhood characteristics, and sociodemographic covariables (*N* = 9,904)

Variables M (SD) or % (*N*)	Total	Black 9.25%(1,579)	Hispanic/Latinx 7.65% (994)	White 83.10% (7,331)	*p*-value
**Loneliness**	1.53 (0.45)	1.62 (0.57)	1.60 (0.50)	1.52 (0.42)	> 0.001
**Perceived institutional discrimination total**	0.18 (0.46)	0.41 (0.94)	0.21 (0.57)	0.15 (0.39)	> 0.001
**Stressful life events total**	0.18 (0.44)	0.25 (0.71)	0.24 (0.59)	0.17 (0.40)	0.002
**Perceived institutional discrimination: unfairly not hired for a job**					> 0.001
No	90.67% (8854)	84.67% (1300)	91.52% (883)	91.24% (6671)	
Yes	9.33% (832)	15.33% (216)	8.48% (74)	8.76% (542)	
**Perceived institutional discrimination: unfairly prevented from moving into a neighborhood**					> 0.001
No	98.18% (9492)	91.07% (1406)	97.49% (930)	99.01% (7156)	
Yes	1.82% (231)	8.93% (129)	2.51% (27)	0.99% (75)	
**Perceived institutional discrimination: unfairly stopped**,** threatened**,** or abused by the police**					> 0.001
No	93.11% (9078)	82.16% (1292)	89.67% (882)	94.62% (6904)	
Yes	6.89% (641)	17.84% (242)	10.33% (75)	5.38% (324)	
**Stressful life event: moving to worse residence/ neighborhood**					0.004
No	97.26% (9523)	94.73% (1482)	97.50% (938)	97.52% (7103)	
Yes	2.74% (242)	5.27% (62)	2.50% (29)	2.48% (151)	
**Stressful life event: being robbed or having home burglarized**					0.005
No	94.93% (9285)	93.12% (1445)	92.57% (893)	95.34% (6947)	
Yes	5.07% (486)	6.88% (100)	7.43% (76)	4.66% (310)	
**Stressful life event: unemployed and looking for a job**					0.002
No	89.34% (8811)	86.75% (1328)	84.94% (805)	90.02% (6678)	
Yes	10.66% (931)	13.25% (214)	15.06% (154)	9.98% (563)	
**Neighborhood discohesion**	1.51 (1.32)	2.29 (1.99)	1.96 (1.74)	1.38 (1.16)	> 0.001
**Neighborhood disorder**	1.45 (1.35)	2.34 (2.08)	1.97 (1.75)	1.31 (1.18)	> 0.001
**Neighborhood unsafety**	1.47 (1.54)	2.46 (2.39)	2.11 (2.06)	1.30 (1.34)	> 0.001
**Neighborhood uncleanliness**	1.44 (1.42)	2.23 (2.23)	1.83 (1.82)	1.31 (1.26)	> 0.001
**Urbanicity**					0.050
Non-Urban	50.64% (4906)	37.28% (549)	45.06% (406)	52.64% (3951)	
Urban	49.36% (4893)	62.72% (1020)	54.94% (577)	47.36% (3296)	
**Region**					> 0.001
Northeast	16.40% (1442)	15.33% (241)	10.19% (104)	17.10% (1097)	
Midwest	27.43% (2548)	17.33% (298)	4.16% (41)	30.70% (2209)	
South	35.93% (3994)	59.00% (922)	44.94% (445)	32.54% (2627)	
West	20.23% (1919)	8.34% (118)	40.72% (403)	19.66% (1398)	
**Gender**					0.002
Men	45.39% (3973)	38.44% (514)	44.93% (408)	46.21% (3051)	
Women	54.61% (5931)	61.56% (1065)	55.07% (586)	53.79% (4280)	
**Age**	64.29 (9.51)	63.11 (11.62)	62.93 (10.23)	64.55 (9.07)	0.019
**Log income**	4.66 (0.62)	4.31 (1.01)	4.23 (1.16)	4.74 (0.49)	> 0.001
**Years of education**	13.36 (2.81)	12.40 (3.63)	10.19 (4.98)	13.76 (2.26)	> 0.001
**Coupled**					> 0.001
No	31.05% (3273)	56.04% (867)	32.55% (293)	28.13% (2113)	
Yes	68.95% (6631)	43.96% (712)	67.45% (701)	71.87% (5218)	
**Employment status**					> 0.001
Not working	51.41% (5918)	58.84% (925)	56.12% (570)	50.14% (4423)	
Working	48.59% (3986)	41.16% (654)	43.88% (424)	49.86% (2908)	
**Wave**					0.956
2010	50.17% (5688)	50.31% (876)	50.74% (536)	50.10% (4276)	
2012	49.83% (4216)	49.69% (703)	49.26% (458)	49.90% (3055)	

**Note:** Survey weights were applied to all percentages and frequencies are unweighted. Percentages and N are presented for categorical variables while means and standard deviations are presented for continuous variables

Significant differences were observed for most study variables by race/ethnicity. Black older adults reported the highest levels of loneliness (M = 1.62, SD = 0.57), followed by Hispanic/Latinx older adults (M = 1.60, SD = 0.50). Black older adults also had the highest average score for total perceived institutional discrimination (M = 0.41, SD = 0.94) and total stressful life events (M = 0.25, SD = 0.71), followed by Hispanic/Latinx older adults (M = 0.21, SD = 0.57 and M = 0.24, SD = 0.59, respectively). For neighborhood stressors, Black older adults had the highest rates of neighborhood discohesion (M = 2.29, SD = 1.99), disorder (M = 2.34, SD = 2.08), unsafety (M = 2.46, SD = 2.39), and uncleanliness (M = 2.23, SD = 2.23), than compared to Hispanic/Latinx older adults and White older adults.

For each of the perceived institutional discrimination events, Black older adults had the highest rates of reporting unfairly not hired for a job (15.33%), being prevented from moving into a neighborhood (8.93%), and receiving unfair treatment during police encounters (17.84%). Hispanic/Latinx older adults reported higher rates of being prevented from moving into a neighborhood (2.51%) and unfair treatment during police encounters (10.33%) than White older adults (0.99% and 5.38% respectively). White older adults reported higher levels of discrimination in job hiring (8.76%) compared to Hispanic/Latinx older adults (8.48%). For individual stressful life events, Black older adults were the most likely to report moving to a worse neighborhood or residence (5.27%), compared to Hispanic/Latinx older adults (2.50%) and White older adults (2.48%). Hispanic/Latinx older adults were most likely to be robbed/burglarized (7.43%) and unemployed and looking for work (15.06%), followed by Black older adult (6.88% and 13.25%, respectively) and White older adults (4.66% and 9.98%, respectively).

### Multivariable results

The first set of multivariable regression models controlling for all study covariables are presented in Table 2. These models examine the associations between the total number of institutional discrimination experiences, stressful life events, and neighborhood factors of disorder and discohesion on loneliness four years later. These models are also stratified by race/ethnicity. Results within each racial/ethnic group are as follows:

**Table 2:** Race/ethnicity stratified multivariable ordinary least squares regression model of loneliness on perceived institutional discrimination, stressful life events, neighborhood characteristics, and sociodemographic covariables (*N* = 9,904)

Variables	Black b [95% CI]	Hispanic/Latinx b [95% CI]	White b [95% CI]
**Perceived institutional discrimination total**	0.04* [0.01, 0.08]	0.03 [-0.07, 0.14]	0.08*** [0.05, 0.11]
**Stressful life events total**	0.06** [0.02, 0.10]	-0.02 [-0.07, 0.03]	0.05** [0.01, 0.09]
**Neighborhood discohesion**	0.07*** [0.05, 0.10]	0.09*** [0.06, 0.11]	0.12*** [0.10, 0.13]
**Neighborhood disorder**	-0.02 [-0.04, 0.01]	-0.03 [-0.06, 0.00]	-0.04*** [-0.05,-0.02]
**Urban**			
Not urban (ref.)	-----	-----	-----
Urban	-0.02 [-0.09, 0.04]	-0.06 [-0.13, 0.02]	-0.03* [-0.05, -0.00]
**Region**			
South (ref.)	-----	-----	-----
Northeast	0.02 [-0.08, 0.12]	-0.03 [-0.17, 0.12]	-0.02 [-0.06, 0.02]
Midwest	0.03 [-0.03, 0.08]	-0.08 [-0.22, 0.05]	0.02 [-0.01, 0.05]
West	0.00 [-0.09, 0.10]	-0.01 [-0.09, 0.07]	0.01 [-0.02, 0.04]
**Gender**			
Men (ref.)	-----	-----	-----
Women	-0.05 [-0.10, 0.01]	-0.01 [-0.09, 0.07]	-0.06*** [-0.09, -0.04]
**Age**	-0.00 [-0.01, 0.00]	0.00 [-0.01, 0.01]	-0.00** [-0.00, -0.00]
**Income**	-0.04* [-0.07, -0.00]	-0.03 [-0.06, 0.01]	-0.08** [-0.12, -0.04]
**Years of education**	-0.01* [-0.02, -0.00]	-0.00 [-0.01, 0.01]	-0.02*** [-0.02, -0.01]
**Couple status**			
Not coupled (ref.)	-----	-----	-----
Coupled	-0.07* [-0.13, -0.00]	-0.10* [-0.17, -0.02]	-0.07*** [-0.10, -0.04]
**Employment status**			
Not working (ref.)	-----	-----	-----
Working (ref.)	-0.07* [-0.13, -0.01]	-0.13** [-0.22, -0.04]	-0.06** [-0.10, -0.02]
**Wave**			
2010 (ref.)	-----	-----	-----
2012	-0.01 [-0.06, 0.05]	0.03 [-0.05, 0.12]	-0.01 [-0.03, 0.01]
**Constant**	2.01*** [1.71, 2.32]	1.75*** [1.32, 2.18]	2.26*** [2.05, 2.47]

**Note:** Regression coefficients and 95% confidence intervals are presented. * indicates statistical significance a the 0.05 level, ** indicates statistical significance at the 0.01 level, and *** indicates statistical significance at the 0.001 level

#### ***Black older adults***

Both total perceived institutional discrimination (b = 0.04, 95% CI: 0.01, 0.08) and stressful life events (b = 0.06, 95% CI: 0.02, 0.10) were associated with greater loneliness. Additionally, greater neighborhood discohesion was associated with greater loneliness (b = 0.07, 95% CI: 0.05, 0.10).

#### ***Hispanic/Latinx older adults***

Greater neighborhood discohesion was associated with greater loneliness (b = 0.09, 95% CI: 0.06, 0.11). No other associations were statistically significant for this group of adults.

#### ***White older adults***

Total perceived institutional discrimination (b = 0.08, 95% CI: 0.05, 0.11) and stressful life events (b = 0.05, 95% CI: 0.02, 0.09) were significantly associated with loneliness. For neighborhood stressors, higher neighborhood discohesion (b = 0.12, 95% CI: 0.10, 0.13) was associated with greater loneliness while higher neighborhood disorder (b=-0.04, 95% CI: -0.05, -0.02) was associated with less loneliness.

The second set of fully adjusted multivariable regression models is presented in Table 3. These models examine the relationships between each of the individual items for perceived institutional discrimination and stressful life events, neighborhood discohesion, and neighborhood disorder parsed out as neighborhood unsafety and uncleanliness on loneliness four years later. These models are also stratified by race/ethnicity.

**Table 3:** Race/ethnicity stratified multivariable ordinary least squares regression model of loneliness on individual perceived institutional discrimination events, individual stressful life events, neighborhood characteristics, and sociodemographic covariables (*N* = 9,904)

Variables	Black b [95% CI]	Hispanic/Latinx b [95% CI]	White b [95% CI]
**Perceived institutional discrimination: unfairly not hired for a job**			
No (ref.)	-----	-----	-----
Yes	0.05 [-0.03, 0.14]	-0.02 [-0.15, 0.11]	0.08** [0.03, 0.13]
**Perceived institutional discrimination: unfairly prevented from moving into a neighborhood**			
No (ref.)	-----	-----	-----
Yes	0.02 [-0.08, 0.12]	0.28* [0.06, 0.50]	-0.02 [-0.15, 0.12]
**Perceived institutional discrimination: unfairly stopped**,** threatened**,** or abused by the police**			
No (ref.)	-----	-----	-----
Yes	0.07 [-0.03, 0.16]	0.02 [-0.13, 0.17]	0.10** [0.03, 0.17]
**Stressful life event: moving to a worse residence/neighborhood**			
No (ref.)	-----	-----	-----
Yes	0.12* [0.01, 0.23]	0.03 [-0.13, 0.19]	0.11* [0.00, 0.21]
**Stressful life event: being robbed or having home burglarized**			
No (ref.)	-----	-----	-----
Yes	0.14** [0.04, 0.25]	-0.12* [-0.23, -0.02]	0.07 [-0.02, 0.16]
**Stressful life event: unemployed and looking for work**			
No (ref.)	-----	-----	-----
Yes	-0.02 [-0.11, 0.07]	0.01 [-0.08, 0.11]	0.02 [-0.02, 0.07]
**Neighborhood discohesion**	0.07*** [0.05, 0.10]	0.08*** [0.06, 0.11]	0.12*** [0.10, 0.13]
**Neighborhood unsafety**	-0.02 [-0.03, 0.00]	0.00 [-0.02. 0.03]	-0.02* [-0.03, -0.00]
**Neighborhood uncleanliness**	0.00 [-0.02, 0.03]	-0.04* [-0.06, -0.01]	-0.02** [-0.03, -0.00]
**Urban**			
Not urban (ref.)	-----	-----	-----
Urban	-0.02 [-0.09, 0.04]	-0.05 [-0.12, 0.02]	-0.03 [-0.05, 0.00]
**Region**			
South (ref.)	-----	-----	-----
Northeast	0.02 [-0.08, 0.11]	-0.04 [-0.18, 0.11]	-0.02 [-0.06, 0.02]
Midwest	0.02 [-0.03, 0.08]	-0.09 [-0.22, 0.04]	0.02 [-0.01, 0.05]
West	0.01 [-0.09, 0.11]	-0.02 [-0.10, 0.06]	0.01 [-0.02, 0.03]
**Gender**			
Men (ref.)	-----	-----	-----
Women	-0.04 [-0.10, 0.02]	-0.01 [-0.09, 0.08]	-0.07*** [-0.09, -0.04]
**Age**	-0.00 [-0.01, 0.00]	-0.00 [-0.01, 0.01]	-0.00** [-0.00, -0.00]
**Income**	-0.04* [-0.07, -0.01]	-0.02 [-0.06, 0.02]	-0.08*** [-0.12, -0.04]
**Years of education**	-0.01* [-0.02, -0.00]	-0.00 [-0.01, 0.01]	-0.02*** [-0.02, -0.01]
**Couple Status**			
Not coupled (ref.)	-----	-----	-----
Coupled	-0.07* [-0.13, -0.00]	-0.11** [-0.18, -0.03]	-0.07*** [-0.10, -0.04]
**Employment status**			
Not working (ref.)	-----	-----	-----
Working (ref.)	-0.07* [-0.13, -0.01]	-0.12* [-0.22, -0.03]	-0.06** [-0.10, -0.03]
**Wave**			
2010 (ref.)	-----	-----	-----
2012	-0.01 [-0.07, 0.04]	0.03 [-0.05, 0.11]	-0.01 [-0.03, 0.01]
**Constant**	2.03*** [1.71, 2.35]	1.73*** [1.29, 2.17]	2.26*** [2.05, 2.47]

**Note:** Regression coefficients and 95% confidence intervals are presented. * indicates statistical significance a the 0.05 level, ** indicates statistical significance at the 0.01 level, and *** indicates statistical significance at the 0.001 level

#### ***Black older adults***

Moving into a worse neighborhood or residence (b = 0.12, 95% CI: 0.01, 0.23) and being robbed/burglarized (b = 0.14, 95% CI: 0.04, 0.23) was significantly linked to greater loneliness. Additionally, higher neighborhood discohesion was associated with higher loneliness (b = 0.07, 95% CI: 0.05, 0.10).

#### ***Hispanic/Latinx older adults***

Experiencing discrimination in not being able to move into a neighborhood (b = 0.28, 95% CI: 0.06, 0.50) was significantly associated with greater loneliness while being robbed or having one’s home burglarized (b=-0.12, 95% CI: -0.23, -0.02) was associated with less loneliness. Higher neighborhood discohesion was significantly associated with more loneliness (b = 0.08, 95% CI: 0.06, 0.11), whereas greater uncleanliness was associated with less loneliness (b=-0.04, 95% CI: -0.06, -0.01).

#### ***White older adults***

Discrimination in job hiring (b = 0.08, 95% CI: 0.03, 0.13), discrimination from police encounters (b = 0.10, 95% CI: 0.03, 0.17), and moving into a worse neighborhood or residence (b = 0.11, 95% CI: 0.00, 0.21) were all significantly associated with more loneliness. For neighborhood stressors, higher neighborhood discohesion was associated with more loneliness (b = 0.12, 95% CI: 0.10, 0.13), whereas greater neighborhood unsafety (b=-0.02, 95% CI: -0.03, 0.00) and higher neighborhood uncleanliness (b=-0.02, 95% CI: -0.03, 0.00) were associated with less loneliness.

## Discussion

This study provides unique and important contributions to the literature by examining the relationships between loneliness and multiple stressors spanning the domains of institutional discrimination (e.g., employment, policing, housing), stressful life events (e.g., moving to a worse neighborhood, being robbed, being unemployed), and neighborhood characteristics in a nationally representative sample of racially and ethnically diverse US older adults. Overall, we found racial/ethnic differences in reported experiences of each of the stressors and loneliness as well as in the associations between stressors and loneliness. Our study extends the existing loneliness literature in that these specific stress-loneliness pathways have not been adequately examined in previous research.

In the bivariate analyses, Black older adults reported the highest perceived institutional discrimination and stressful life event totals, followed by Hispanic/Latinx older adults and White older adults. These patterns persisted when examining individual measures included in the total scores for discrimination and stressful life events, where Black older adults and Hispanic/Latinx older adults were more likely to report these experiences than White older adults. The only exception was unfairly not being hired for a job, which was reported by a larger proportion of Black older adults followed by White older adults and Hispanic/Latinx older adults. Moreover, compared to White older adults, Black older adults and Hispanic/Latinx older adults reported worse perceptions of their neighborhoods regarding safety, cleanliness, and social cohesion. These findings align with previous research that demonstrates that Black and Latinx communities experience greater levels of stressors including discrimination, stressful life events, and worse neighborhood contexts than White communities [20, 59, 61, 76, 77]. Additionally, we found that both Black and Hispanic/Latinx older adults reported higher levels of loneliness than White older adults. There have been mixed findings regarding loneliness by race/ethnicity; however, the results of our study align with the growing number of studies which demonstrate greater loneliness among Black older adults and Hispanic/Latinx older adults than White older adults [17, 18, 59]. 

Our multivariate analyses found that greater total perceived institutional discrimination and stressful life events were significantly associated with greater loneliness among both Black and White older adults. When we examined individual indicators of discrimination and stressful life events by race/ethnicity, we found that White older adults had *more* factors that were significantly associated with loneliness than Black and Hispanic/Latinx older adults, including being unfairly not hired for a job, being unfairly stopped, threatened, or abused by the police, and moving to a worse residence/neighborhood. In contrast, only being robbed or burglarized and unfairly prevented from moving into a neighborhood was significantly associated with greater loneliness for Black older adults and Hispanic/Latinx older adults, respectively. In general, our findings are similar to other proposed pathways of loneliness, as Lim and colleagues [64] and Elder and Retrum [11] have postulated that significant stressful life events and/or major transitions can contribute to the development of loneliness.

Nevertheless, we found a greater number of statistically significant stressor-loneliness relationships for White older adults compared to Black older adults and Hispanic/Latinx older adults (three significant relationships with individual stressors compared to one each for Black older adults and Hispanic/Latinx older adults, respectively). Moreover, White older adults also had the lowest number of stressors of all three racial/ethnic groups. What this demonstrates is the importance of stress appraisal, potentially through racial socialization at earlier ages [76]. These findings are similar to those of previous work by Brown et al. [76]., which found that Black and Latinx older adults experience more stressors than White older adults but are less likely to report them as distressing. More specifically, previous research has found that White people may be more vulnerable to worse health outcomes stemming from stressful life events compared to Black and Hispanic/Latinx adults due to the fact that these racial/ethnic groups frequently experience greater exposure to institutional discrimination and stressful life events from earlier ages (e.g., youth). Hence, Black older adults and Hispanic/Latinx older adults may have had more opportunities to develop effective coping skills and ability to leverage resources to undertake these stressful life situations in comparison to White older adults [33, 78]. Mechanistically, loneliness may be generated from stressful experiences through maladaptive social cognitions. Said simply, this is when individuals perceive their environment to be threatening and dangerous to them [55, 56]. In comparing White to racially/ethnically minoritized populations, what may seem as stressful and threatening for White older adults may be viewed as insignificant for Black older adults and Hispanic/Latinx older adults given their development of coping skills and resources to handle similar situations earlier in life.

Another potential explanation for our findings may due to the cultural background and heritage of racially/ethnically minoritized populations, which could play a significant role in preventing loneliness after experiencing discriminatory and stressful life events for Black and Hispanic/Latinx older adults. For example, previous research has found fictive kinship is an important cultural component to Black Americans. Fictive kin are defined as individuals who are not related to each other biologically, but still share close bonds/relationships and carry out functions that are often associated with family, including the provision of social support [79–81]. Previous studies of fictive kin have found Black American older adults are more likely to have fictive kin than White older adults [81]. Fictive kin, therefore, may act as a buffer for reappraising stressful life events so they do not influence feelings of loneliness. Similarly, familism (or familismo) is a cultural tradition in Hispanic/Latinx communities. Familism is described as a process in which individuals care for, are dedicated, have loyalty, and maintain close relationships with both close and extended family members [82, 83]. Previous studies have found identifying as Latinx predicted a stronger association with familism; furthermore, familism is also associated with lower loneliness [82]. Similar to fictive kin for Black older adults, familism may act as a buffer for reducing the impact of perceived institutional discrimination, stressful life events, and poor perceived neighborhood characteristics on loneliness among Hispanic/Latinx older adults.

Interestingly, we also found being robbed or burglarized is associated with less subsequent loneliness among Hispanic/Latinx older adults. This finding is contradictory to our expected outcome that being robbed or burglarized would be associated with greater subsequent loneliness. Nevertheless, we think this finding may also illustrate the mobilization of social support and social networks among Hispanic/Latinx older adults. Social support and network mobilization is a coping strategy in which an individual actively calls on members of their social network to help them process and cope with stressful life events [84–88]. We believe Hispanic/Latinx older adults who were robbed or burglarized subsequently engaged with members of their social networks to increase their levels of social support, which also helped these individuals feel loved and cared for after this traumatic event. This support mobilization would also help strengthen the bonds between the individual and members of their social network which may have resulted in a significant decrease in loneliness, so much so that Hispanic/Latinx older adults who were robbed/burglarized report less loneliness than Hispanic/Latinx older adults who were not robbed/burglarized.

In our study we find multiple stressors were associated with loneliness among White older adults including being unfairly not hired for a job, unfairly stopped by the police, and moving into a worse residence/neighborhood. For White older adults, these events may be new experiences and may also be linked to significant life transitions that are also correlated with loneliness [11, 64]. A recent study found older adults who are movers tend to be renters, live alone, and were also more likely to have lower incomes and higher housing costs in comparison to non-movers; [89] additionally, these factors are all correlated with loneliness. The process of retirement may also lead to moving into different neighborhoods for many older adults. Previous research has found that White residents prefer to live in communities where there is a majority of White residents while Black residents prefer to live in racially integrated neighborhoods [90, 91]. For White older adults, moving into a worse neighborhood or residence may be reflective of the racial composition of a neighborhood (e.g., greater racial integration) and hence, there may be fewer White older adults living around them and in turn, contribute to their loneliness.

For all racial/ethnic groups, perceiving more neighborhood discohesion was associated with greater loneliness. Moreover, nuanced differences were found in the association between perceived neighborhood characteristics and loneliness across groups. As an example, White older adults who perceived their neighborhood as being more unsafe and unclean had less loneliness. Moreover, greater perceived uncleanliness was associated with less loneliness among Hispanic/Latinx older adults. Perceived neighborhood disorder, in total or by individual safety and cleanliness indicators, was not associated with loneliness among Black older adults.

Our findings align with previous research indicating that having positive relationships with neighbors protected against loneliness [48]. For older adults, neighborhood social cohesion may be particularly salient for loneliness due to older adults potentially having to rely on their neighbors for support and assistance with daily tasks, especially if family members are not close by. However, previous research indicates that a greater proportion of Black respondents reported not having friends in the neighborhood but having family residing in their neighborhood [59], which adds nuance to racial/ethnic group specific relationship dynamics with others in one’s neighborhood. Previous research has demonstrated that during times of crisis, neighborhoods and communities have played a critical role [92]. Specifically, across several studies, after major destruction from hurricanes (e.g., Hurricane Sandy, Maria, Katrina), residents have relied on their neighbors, faith-based organizations, and other sources of informal support in their communities in undertaking post recovery efforts [58, 93, 94]. These studies demonstrate that Black and Hispanic/Latinx communities often are unable to rely on formal sources of support. This is especially given racially/ethnically minoritized communities often have constrained resources, infrastructure, and continued disinvestment. Hence, neighborhoods may play an important function in the promotion of social interactions and in the establishment of critical supportive connections for older adults [92]. 

Further, we found that greater perceived neighborhood unsafety was associated with lower loneliness scores among White older adults, and greater perceived neighborhood uncleanliness was associated with lower loneliness among both White and Hispanic/Latinx older adults. These results were unexpected. Previous research indicates that more negative perceptions of the neighborhood environment (i.e., poorer social cohesion, fewer resources for physical activity/walking, less safety) were associated with greater loneliness [49]. However, the sample from Kowitt and colleagues’ study [49] was comprised of White and Black American older adults in North Carolina, and analyses were not stratified by race/ethnicity to obtain information on any racial variation on how perceived neighborhood context is associated with loneliness. One potential reason for our findings is that perceived neighborhood safety and cleanliness are proxies of urban residence, whereby living near others may reduce loneliness. Given that greater social cohesion was a consistent predictor of lower loneliness scores in our sample, the benefits conferred through social connections may outweigh any stress associated with neighborhood disorder. For example, Hispanic/Latinx enclaves may be under-resourced due to structural racism and thus be characterized as having more physical disorder and uncleanliness. However, living in communities with others with shared cultural backgrounds can lead to closer social connections and increased levels of social support, which can minimize feelings of loneliness [82, 95]. Recent studies have found that neighborhood social cohesion was more salient for better cardiovascular health outcomes for Black women than Black men [96] and improved perceived neighborhood safety was associated with body mass index among Black women [97]. Though we did not test differences by gender, we recommend future studies to explore these relationships stratified by gender in understanding implications for loneliness.

### Limitations

Perceived measures of institutional racism, stressful life events, and neighborhood characteristics were used in this study, which may be subject to self-report bias. However, perceived neighborhood measures have been found to be salient for health and loneliness [98–101], with some evidence demonstrating that perceived neighborhood factors are more salient than objective neighborhood indicators [54]. Moreover, future research should assess the independent and interactive effects of objective and perceived measures on loneliness. Additionally, given the varied relationships between stressors and loneliness across racial/ethnic groups, future studies may include additional neighborhood context measures, both objective and perceived, to further disentangle how structural racism, discrimination, and stressors affect loneliness and subsequent health outcomes for Black and Hispanic/Latinx older adults [102]. The present study used measures of perceived institutional discrimination and stressful life events from one point in time (2010–2012) due to study limitations (e.g., this data was not collected for every HRS cycle), and hence, did not allow for assessing the association between these stressors and loneliness over time. Future research should include multiple waves of data to examine the various perceived institutional discrimination, stressful life events, and perceived neighborhood characteristics shape trajectories of loneliness across racial/ethnic groups. Moreover, studies should aim to understand how these and other stressful experiences over the life course may influence loneliness in older adulthood.

Our study also does not capture the full dynamics of housing discrimination and gentrification. For example, previous research has found substantial racial/ethnic discrimination in both purchasing a home and rental housing markets [103, 104]. Given these findings, this may lead to increased feelings of loneliness among racially/ethnically minoritized populations. In a similar vein, neighborhood gentrification has been associated with greater loss of social networks and greater social isolation, especially among Black older adults [105, 106], which may lead to greater feelings of loneliness. Merging these areas of research would greatly extend our knowledge of how structural racism, stressful life events, and changing neighborhood characteristics influence loneliness among racially/ethnically minoritized older adults.

Further, our study did not assess how the relationships examined may vary by socioeconomic status (SES), which should be addressed in future work. Lower SES may be related with greater loneliness compared to higher SES as supported by previous research showing that individuals with lower SES often have smaller social networks and limited access to networks and resources to socially engage with others [12, 15]. Assessing interactions between race/ethnicity and SES will increase our understanding of how discrimination and other stressors are associated with loneliness within subgroups of older adults, thus identifying those most at risk for loneliness and related health consequences. Despite some of these aforementioned limitations, our study provides novel contributions to the literature in exploring perceived discrimination, stressful life events, and neighborhood factors, which have not been extensively examined in relation to loneliness, as well as understanding which of these stressors are most associated with loneliness by race/ethnicity using a representative sample of US older adults.

### Research and practice implications

Our study has important implications for research and practice. First, our study findings demonstrate that both Black and Hispanic/Latinx older adults experience greater stressors; however, these stressors have differential implications for loneliness in comparison to White older adults. Additionally, it is critical to note that stressors are interlinked and dynamic in nature [107, 108]. For example, it is plausible that perceptions of neighborhood safety may be linked to previous stressful experiences such as being robbed or burglarized, and in combination, may have enhanced effects for loneliness. Our study provides an initial understanding of critical stressors that are salient for loneliness by race/ethnicity and we recommend future investigations to build upon our findings to elucidate multiplicative effects of stressors for loneliness, especially among Black and Hispanic/Latinx older adults. We invite future research to investigate the role of multiple stressors interlinked with both perceived and objective neighborhood factors for loneliness.

Additionally, investigations employing qualitative methodologies would enhance our understanding of how loneliness operates among different racial/ethnic groups and in different settings/contexts among older adults. Our findings demonstrate that across several stressors, perceived institutional discrimination and stressful life events were less likely to influence loneliness among Hispanic/Latinx older adults; however, given the nature of our study, we are unable to assess how or why these factors do not influence loneliness uniformly across racial/ethnic groups, or if there are critical periods for when these stressors may be most consequential for loneliness. Furthermore, qualitative data would provide additional insight into the association between perceived neighborhood characteristics and loneliness. This is especially important given our study found greater neighborhood discohesion was consistently related to greater loneliness across all racial/ethnic groups, and because older adults spend substantially more time in their neighborhood communities in comparison to other age groups [42]. 

The results of our study can also inform future interventions. It will be important to customize interventions across diverse groups of older adults to ensure that they are more effective at decreasing loneliness for these groups, especially given our findings illustrating that different stressors were associated with loneliness by race/ethnicity. Said another way, what works for White older adults may not be applicable for Black and Hispanic/Latinx older adults. To further add to this complexity, recent reviews of loneliness note the importance of utilizing different types of intervention strategies to decrease loneliness [109]. It will be vital to develop and/or strengthen cross-sector collaborations across different agencies and organizations working together with the overarching goal to ameliorate loneliness and improve social connectedness [8]. This can be done by fostering relationships with local churches and religious organizations, senior centers, and community affinity groups at the neighborhood level. This may contribute to increased feelings of trust and safety for older adults within their neighborhoods and enhance their quality of relationships within their communities and subsequently, reduce loneliness. As noted in a recent Campbell Collaboration systematic review on interventions to reduce loneliness, the authors noted that there are relatively few interventions for loneliness which address structural changes in neighborhood environments [109], with greater attention needed in this area. Beyond strengthening relationships between community members, consistent and long-term investments in communities/environments may have the strongest potential effects in decreasing loneliness among older adults.

## Conclusion

Our study contributes to the emerging literature that provides evidence of how perceived institutional discrimination, stressful life events, and neighborhood characteristics contribute to loneliness among older adults in the US. We also found that these stressors contribute to loneliness with distinct differences across groups. Black older adults experienced the greatest number of stressors; however, unexpectedly, all stressors had greater effects for loneliness for White older adults illustrating nuanced and complex ways these stressors contribute to loneliness and the differing stress appraisal processes across groups. With increasing projections of the US older adult population, including this segment of population becoming more racially and ethnically diverse, it has become increasingly vital for more research to examine and understand how multiple stressors contribute to loneliness among older adults. Our findings can inform future interventions and policies focused on reducing exposures to various structural stressors, in tandem with strengthening the quality of social relationships among those most vulnerable to loneliness.

## Electronic supplementary material

Below is the link to the electronic supplementary material.

Supplementary Material 1

## Data Availability

Data from the Health and Retirement Study is available for download from the following website: https://hrs.isr.umich.edu/about.

## Abbreviations

WOAs: White older adults
BOAs: Black older adults
HOAs: Hispanic/Latinx older adults
PID: Perceived institutional discrimination
SLE: Stressful life events
PNC: Perceived neighborhood characteristics

## References

[1] Perlman D, Peplau LA. Toward a social psychology of loneliness. Pers Relatsh. 1981;3:31–56.

[2] Courtin E, Knapp M. Social isolation, loneliness and health in old age: a scoping review. Health Soc Care Community. 2017;25(3):799–812. 10.1111/hsc.12311. 26712585 10.1111/hsc.12311

[3] Ong AD, Uchino BN, Wethington E. Loneliness and health in older adults: a Mini-review and Synthesis. Gerontology. 2016;62(4):443–9. 10.1159/000441651. 26539997 10.1159/000441651 PMC6162046

[4] Luo Y, Hawkley LC, Waite LJ, Cacioppo JT. Loneliness, health, and mortality in old age: A national longitudinal study. *Soc Sci Med*. Published online. 2012. 10.1016/j.socscimed.2011.11.028 10.1016/j.socscimed.2011.11.028 PMC3303190 22326307

[5] National Academies of Sciences, Engineering, and, Medicine. Social isolation and loneliness in older adults: opportunities for the Health Care System. National Academies; 2020. 32510896

[6] Taylor HO, Cudjoe TKM, Bu F, Lim MH. The state of loneliness and social isolation research: current knowledge and future directions. BMC Public Health. 2023;23(1):1049. 10.1186/s12889-023-15967-3. 37264355 10.1186/s12889-023-15967-3 PMC10233527

[7] US Department of Health and Human Services. Our Epidemic of Loneliness and Isolation: The U.S. Surgeon General’s Advisory on the Healing Effects of Social Connection and Community. 2023. Accessed June 11, 2023. https://www.hhs.gov/sites/default/files/surgeon-general-social-connection-advisory.pdf 37792968

[8] World Health Organization. Social isolation and loneliness among older people: advocacy brief. Published online 2021.

[9] Vespa JE, Medina L, and Armstrong DM,. Demographic Turning Points for the United States: Population Projections for 2020 to 2060. Current Population Reports, P25-1144, U.S. Census Bureau, Washington, DC. 2020.

[10] Hawkley LC, Buecker S, Kaiser T, Luhmann M. Loneliness from young adulthood to old age: explaining age differences in loneliness. Int J Behav Dev. 2022;46(1):39–49. 10.1177/0165025420971048. 35001993 10.1177/0165025420971048 PMC8734589

[11] Elder K, Retrum J. Framework for isolation in adults over 50. AARP Foundation; 2012.

[12] Finlay JM, Meltzer G, Cannon M, Kobayashi LC. Aging in Place during a pandemic: Neighborhood Engagement and environments since the COVID-19 pandemic onset. Gerontologist. 2022;62(4):504–18. 10.1093/geront/gnab169. 34788816 10.1093/geront/gnab169 PMC8767892

[13] Goveas JS, Ray RM, Woods NF, et al. Associations between changes in loneliness and Social Connections, and Mental Health during the COVID-19 pandemic: the women’s Health Initiative. J Gerontol Ser A. 2022;77(Supplement1):S31–41. 10.1093/gerona/glab371. 10.1093/gerona/glab371 PMC8754805 34915558

[14] Polenick CA, Perbix EA, Salwi SM, Maust DT, Birditt KS, Brooks JM. Loneliness during the COVID-19 pandemic among older adults with chronic conditions. J Appl Gerontol. 2021;40(8):804–13. 10.1177/0733464821996527. 33641513 10.1177/0733464821996527 PMC8238795

[15] Sol K, Brauer S, Antonucci TC. Longitudinal associations between loneliness and self-rated Health among Black and White older adults. J Gerontol Ser B. 2023;78(4):639–48. 10.1093/geronb/gbac200. 10.1093/geronb/gbac200 PMC10066738 36571393

[16] Finlay JM, Kobayashi LC. Social isolation and loneliness in later life: a parallel convergent mixed-methods case study of older adults and their residential contexts in the Minneapolis metropolitan area, USA. Soc Sci Med. 2018;208:25–33. 10.1016/j.socscimed.2018.05.010. 29758475 10.1016/j.socscimed.2018.05.010

[17] Taylor HO, Nguyen AW. Depressive symptoms and loneliness among Black and White older adults: the moderating effects of race. Innov Aging. 2020;4(5):igaa048. 33354628 10.1093/geroni/igaa048 PMC7739884

[18] Hawkley LC, Hughes ME, Waite LJ, Masi CM, Thisted RA, Cacioppo JT. From Social Structural factors to perceptions of Relationship Quality and loneliness: the Chicago Health, Aging, and Social relations Study. J Gerontol B Psychol Sci Soc Sci. 2008;63(6):S375–84. 19092047 10.1093/geronb/63.6.s375 PMC2769562

[19] Taylor H. Social isolation, loneliness, and Physical and Mental Health among Black Older adults. Annu Rev Gerontol Geriatr. 2022;1123–44. 10.1891/0198-8794.41.123.

[20] Sternthal MJ, Slopen N, Williams DR. RACIAL DISPARITIES IN HEALTH: how much does stress really Matter? Bois Rev Soc Sci Res Race. 2011;8(1):95–113. 10.1017/S1742058X11000087. 10.1017/S1742058X11000087 PMC5993442 29887911

[21] Williams DR, Lawrence JA, Davis BA. Racism and health: evidence and needed research. Annu Rev Public Health. 2019;40(1):105–25. 10.1146/annurev-publhealth-040218-043750. 30601726 10.1146/annurev-publhealth-040218-043750 PMC6532402

[22] Adkins-Jackson PB, Chantarat T, Bailey ZD, Ponce NA. Measuring structural racism: a Guide for epidemiologists and Other Health Researchers. Am J Epidemiol. 2022;191(4):539–47. 10.1093/aje/kwab239. 34564723 10.1093/aje/kwab239 PMC9077112

[23] Bailey ZD, Krieger N, Agénor M, Graves J, Linos N, Bassett MT. Structural racism and health inequities in the USA: evidence and interventions. Lancet. 2017;389(10077):1453–63. 10.1016/S0140-6736(17)30569-X. 28402827 10.1016/S0140-6736(17)30569-X

[24] Chatters LM, Taylor HO, Taylor RJ. Racism and the Life Course: Social and Health Equity for Black American older adults. Public Policy Aging Rep. 2021;31(4):113–8. 10.1093/ppar/prab018. 34691479 10.1093/ppar/prab018 PMC8528194

[25] Blankenship KM, Rosenberg A, Schlesinger P, Groves AK, Keene DE. Structural racism, the Social Determination of Health, and Health inequities: the intersecting impacts of Housing and Mass Incarceration. Am J Public Health. 2023;113(S1):S58–64. 10.2105/AJPH.2022.307116. 36696621 10.2105/AJPH.2022.307116 PMC9877374

[26] Williams DR, Collins C. Racial residential segregation: a Fundamental cause of racial disparities in Health. Public Health Rep. 2001;116(5):404–16. 10.1093/phr/116.5.404. 12042604 10.1093/phr/116.5.404 PMC1497358

[27] Redwood Y, Schulz AJ, Israel BA, Yoshihama M, Wang CC, Kreuter M. Social, Economic, and political processes that create built Environment inequities: perspectives from urban African americans in Atlanta. Fam Community Health. 2010;33(1). http://journals.lww.com/familyandcommunityhealth/Fulltext/2010/01000/Social,_Economic,_and_Political_Processes_That.8.aspx 10.1097/FCH.0b013e3181c4e2d4 20010005

[28] Ross CE, Mirowsky J. Neighborhood disadvantage, disorder, and Health. J Health Soc Behav. 2001;42(3):258–76. 11668773

[29] Williams DR, Mohammed SA. Discrimination and racial disparities in health: evidence and needed research. J Behav Med. 2009;32(1):20–47. 10.1007/s10865-008-9185-0. 19030981 10.1007/s10865-008-9185-0 PMC2821669

[30] McClure E, Feinstein L, Cordoba E, et al. The legacy of redlining in the effect of foreclosures on Detroit residents’ self-rated health. Health Place. 2019;55:9–19. 10.1016/j.healthplace.2018.10.004. 30448354 10.1016/j.healthplace.2018.10.004 PMC6345551

[31] Bhat AC, Almeida DM, Fenelon A, Santos-Lozada AR. A longitudinal analysis of the relationship between housing insecurity and physical health among midlife and aging adults in the United States. SSM - Popul Health. 2022;18:101128. 10.1016/j.ssmph.2022.101128. 35652088 10.1016/j.ssmph.2022.101128 PMC9149198

[32] Mehdipanah R, Bess K, Tomkowiak S, et al. Residential racial and socioeconomic segregation as predictors of housing discrimination in Detroit Metropolitan Area. Sustainability. 2020;12(24). 10.3390/su122410429.

[33] Schulz A, Williams D, Israel B, et al. Unfair treatment, Neighborhood effects, and Mental Health in the Detroit Metropolitan Area. J Health Soc Behav. 2000;41(3):314–32. 10.2307/2676323. 11011507

[34] Hanson A, Hawley Z. Where does racial discrimination occur? An experimental analysis across neighborhood and housing unit characteristics. Reg Sci Urban Econ. 2014;44:94–106. 10.1016/j.regsciurbeco.2013.12.001.

[35] Barr AB, Simons RL, Beach SRH, Simons LG. Racial discrimination and health among two generations of African American couples. Soc Sci Med. 2022;296:114768. 10.1016/j.socscimed.2022.114768. 35168058 10.1016/j.socscimed.2022.114768

[36] Trail TE, Goff PA, Bradbury TN, Karney BR. The costs of racism for marriage: how racial discrimination hurts, and ethnic identity protects, Newlywed Marriages among latinos. Pers Soc Psychol Bull. 2012;38(4):454–65. 10.1177/0146167211429450. 22109252 10.1177/0146167211429450 PMC6623982

[37] Lincoln KD, Chae DH, Stress. Marital satisfaction, and psychological distress among African americans. J Fam Issues. 2010;31(8):1081–105. 10.1177/0192513X10365826.

[38] Murry VM, Brown PA, Brody GH, Cutrona CE, Simons RL. Racial discrimination as a moderator of the links among stress, maternal psychological functioning, and Family relationships. J Marriage Fam. 2001;63(4):915–26. 10.1111/j.1741-3737.2001.00915.x.

[39] Nguyen AW, Taylor HO, Keith VM, Qin W, Mitchell UA. Discrimination and social isolation among African americans: the moderating role of skin tone. Cultur Divers Ethnic Minor Psychol. Published online 2022. 10.1037/cdp0000569 PMC10225012 36441993

[40] Lee Y, Bierman A. Loneliness as a mediator of Perceived discrimination and depression: Examining Education contingencies. Int J Aging Hum Dev. 2019;89(2):206–27. 10.1177/0091415018763402. 29557191 10.1177/0091415018763402

[41] Chen D, Yang TC. The pathways from perceived discrimination to self-rated health: an investigation of the roles of distrust, social capital, and health behaviors. Soc Sci Med. 2014;104:64–73. 10.1016/j.socscimed.2013.12.021. 24581063 10.1016/j.socscimed.2013.12.021 PMC4048349

[42] Glass TA, Balfour JL. Neighborhoods, aging, and functional limitations. In: Kawachi I, Berkman LF, editors. Neighborhoods and health. Oxford University Press; 2003. pp. 303–34.

[43] Pynoos J. The future of housing for the Elderly: four strategies that can make a difference. Public Policy Aging Rep. 2018;28(1):35–8. 10.1093/ppar/pry006.

[44] Manini TM. Mobility decline in Old Age: a time to Intervene. Exerc Sport Sci Rev. 2013;41(1). https://journals.lww.com/acsm-essr/fulltext/2013/01000/mobility_decline_in_old_age__a_time_to_intervene.2.aspx 10.1097/JES.0b013e318279fdc5 PMC3530168 23262463

[45] Charles ST, Carstensen LL. Social and Emotional Aging. Annu Rev Psychol. 2010;61(1):383–409. 10.1146/annurev.psych.093008.100448. 19575618 10.1146/annurev.psych.093008.100448 PMC3950961

[46] Weden MM, Carpiano RM, Robert SA. Subjective and objective neighborhood characteristics and adult health. Soc Sci Med. 2008;66(6):1256–70. 10.1016/j.socscimed.2007.11.041. 18248865 10.1016/j.socscimed.2007.11.041

[47] Bower M, Kent J, Patulny R, et al. The impact of the built environment on loneliness: a systematic review and narrative synthesis. Health Place. 2023;79:102962. 10.1016/j.healthplace.2022.102962. 36623467 10.1016/j.healthplace.2022.102962

[48] Buecker S, Ebert T, Götz FM, Entringer TM, Luhmann M. In a lonely place: investigating Regional differences in loneliness. Soc Psychol Personal Sci. 2021;12(2):147–55. 10.1177/1948550620912881.

[49] Kowitt SD, Aiello AE, Callahan LF, et al. Associations among neighborhood poverty, perceived neighborhood environment, and depressed mood are mediated by physical activity, perceived individual control, and loneliness. Health Place. 2020;62:102278. 10.1016/j.healthplace.2019.102278. 32479356 10.1016/j.healthplace.2019.102278 PMC9540601

[50] Lyu Y, Forsyth A, Planning. Aging, and loneliness: reviewing evidence about built Environment effects. J Plan Lit. 2022;37(1):28–48. 10.1177/08854122211035131.

[51] Yang Y, Xiang X. Examine the associations between perceived neighborhood conditions, physical activity, and mental health during the COVID-19 pandemic. Health Place. 2021;67:102505. 10.1016/j.healthplace.2021.102505. 33454564 10.1016/j.healthplace.2021.102505 PMC9760872

[52] Zaheed AB, Sharifian N, Kraal AZ, Sol K, Hence A, Zahodne LB. Unique effects of Perceived Neighborhood Physical Disorder and Social Cohesion on episodic memory and semantic fluency. Arch Clin Neuropsychol. 2019;34(8):1346–55. 10.1093/arclin/acy098. 30715092 10.1093/arclin/acy098 PMC13745194

[53] Zhang L, Zhou S, Kwan MP. A comparative analysis of the impacts of objective versus subjective neighborhood environment on physical, mental, and social health. Health Place. 2019;59:102170. 10.1016/j.healthplace.2019.102170. 31422227 10.1016/j.healthplace.2019.102170

[54] Wen M, Hawkley LC, Cacioppo JT. Objective and perceived neighborhood environment, individual SES and psychosocial factors, and self-rated health: an analysis of older adults in Cook County, Illinois. Soc Sci Med. 2006;63(10):2575–90. 10.1016/j.socscimed.2006.06.025. 16905230 10.1016/j.socscimed.2006.06.025

[55] Hawkley LC, Cacioppo JT. Loneliness matters: a theoretical and empirical review of consequences and mechanisms. Ann Behav Med. 2010;40(2):218–27. 10.1007/s12160-010-9210-8. 20652462 10.1007/s12160-010-9210-8 PMC3874845

[56] Hawkley LC, Cacioppo JT. Aging and loneliness: Downhill Quickly? Curr Dir Psychol Sci. 2007;16(4):187–91. 10.1111/j.1467-8721.2007.00501.x.

[57] Latham K, Clarke PJ. Neighborhood Disorder, Perceived Social Cohesion, and Social Participation among Older americans: findings from the National Health & Aging trends Study. J Aging Health. 2018;30(1):3–26. 10.1177/0898264316665933. 27566464 10.1177/0898264316665933

[58] Cagney KA, Sterrett D, Benz J, Tompson T. Social Resources and Community Resilience in the wake of Superstorm Sandy. PLoS ONE. 2016;11(8):e0160824. 10.1371/journal.pone.0160824. 27579482 10.1371/journal.pone.0160824 PMC5006987

[59] Massihzadegan S, Stokes JE. Neighborhood Social Cohesion and Loneliness in Mid- and later life: are benefits contingent on Race/Ethnicity or Neighborhood Disorder? J Gerontol Ser B. 2023;78(9):1581–90. 10.1093/geronb/gbad081. 10.1093/geronb/gbad081 37218292

[60] Thierry AD. Association between telomere length and neighborhood characteristics by race and region in US midlife and older adults. Health Place. 2020;62:102272. 10.1016/j.healthplace.2019.102272. 32479352 10.1016/j.healthplace.2019.102272 PMC7266826

[61] Thierry AD, Sherman-Wilkins K, Armendariz M, Sullivan A, Farmer HR. Perceived Neighborhood characteristics and cognitive functioning among diverse older adults: an Intersectional Approach. Int J Environ Res Public Health. 2021;18(5). 10.3390/ijerph18052661. 10.3390/ijerph18052661 PMC7967341 33800952

[62] Hawkley LC, Cacioppo JT. Loneliness and pathways to disease. *Biol Mech Psychosoc Eff Dis Implic Cancer Control*. 2003;17(1, Supplement):98–105. 10.1016/S0889-1591(02)00073-9 10.1016/s0889-1591(02)00073-9 12615193

[63] Kang Jeun, Graham-Engeland JE, Scott S, Smyth JM, Sliwinski MJ. The relationship between loneliness and the experiences of everyday stress and stressor-related emotion. Stress Health. 2023;n/a(n/a). 10.1002/smi.3294. 37526522 10.1002/smi.3294 PMC10830881

[64] Lim MH, Eres R, Vasan S. Understanding loneliness in the twenty-first century: an update on correlates, risk factors, and potential solutions. Soc Psychiatry Psychiatr Epidemiol. 2020;55(7):793–810. 10.1007/s00127-020-01889-7. 32524169 10.1007/s00127-020-01889-7

[65] Wang K, Zhang A, Cuevas AG, De Fries CM, Hinton L, Falcón LM. The Association between post-traumatic stress and depressive symptoms among older puerto ricans in Boston: how does Loneliness Matter? J Aging Health. 2022;34(6–8):786–93. 10.1177/08982643211064123. 34949131 10.1177/08982643211064123

[66] Barjaková M, Garnero A, d’Hombres B. Risk factors for loneliness: a literature review. Soc Sci Med. 2023;334:116163. 10.1016/j.socscimed.2023.116163. 37625251 10.1016/j.socscimed.2023.116163 PMC10523154

[67] Dahlberg L, McKee KJ, Frank A, Naseer M. A systematic review of longitudinal risk factors for loneliness in older adults. Aging Ment Health. 2022;26(2):225–49. 10.1080/13607863.2021.1876638. 33563024 10.1080/13607863.2021.1876638

[68] Fisher GG, Ryan LH. Overview of the Health and Retirement Study and introduction to the Special Issue. Work Aging Retire. 2017;4(1):1–9. 10.1093/workar/wax032. 29423243 10.1093/workar/wax032 PMC5798643

[69] Smith J, Ryan L, Sonnega A, Weir D. *Psychosocial and Lifestyle Questionnaire* 2006–2016.; 2017. https://hrs.isr.umich.edu/sites/default/files/biblio/HRS 2006–2016 SAQ Documentation_07.06.17.pdf.

[70] Russell DW. UCLA Loneliness Scale (Version 3): reliability, validity, and factor structure. J Pers Assess Published Online. 1996. 10.1207/s15327752jpa6601_2. 10.1207/s15327752jpa6601_2 8576833

[71] Williams DR, Yu Y, Jackson JS, Anderson NB. Racial Differences in Physical and Mental Health: Socio-economic status, stress and discrimination. J Health Psychol. 1997;2(3):335–51. 10.1177/135910539700200305. 22013026 10.1177/135910539700200305

[72] Pascoe EA, Richman LS. Perceived discrimination and health: a meta-analytic review. Psychol Bull. 2009;135(4):531. 19586161 10.1037/a0016059 PMC2747726

[73] Albert MA, Cozier Y, Ridker PM, et al. Perceptions of Race/Ethnic discrimination in relation to Mortality among Black women: results from the Black women’s Health Study. Arch Intern Med. 2010;170(10):896–904. 10.1001/archinternmed.2010.116. 20498418 10.1001/archinternmed.2010.116 PMC3020569

[74] Taylor HO. Social isolation’s influence on loneliness among older adults. Clin Soc Work J. 2020;48(1):140–51. 10.1007/s10615-019-00737-9. 33343042 10.1007/s10615-019-00737-9 PMC7747874

[75] White IR, Royston P, Wood AM. Multiple imputation using chained equations: issues and guidance for practice. Stat Med. 2011;30(4):377–99. 10.1002/sim.4067. 21225900 10.1002/sim.4067

[76] Brown LL, Mitchell UA, Ailshire JA. Disentangling the stress process: Race/Ethnic differences in the exposure and Appraisal of Chronic stressors among older adults. J Gerontol Ser B. 2020;75(3):650–60. 10.1093/geronb/gby072. 10.1093/geronb/gby072 PMC7328036 29878196

[77] Mujahid MS, Roux AVD, Cooper RC, Shea S, Williams DR. Neighborhood stressors and Race/Ethnic differences in hypertension prevalence (the multi-ethnic study of atherosclerosis). Am J Hypertens. 2011;24(2):187–93. 10.1038/ajh.2010.200. 20847728 10.1038/ajh.2010.200 PMC3319083

[78] Thomas AJ, Speight SL, Witherspoon KM. Racial socialization, racial identity, and race-related stress of African American parents. Fam J. 2010;18(4):407–12. 10.1177/1066480710372913.

[79] Taylor RJ, Chatters LM, Woodward AT, Brown E. Racial and ethnic differences in Extended Family, Friendship, Fictive Kin, and Congregational Informal Support Networks. Fam Relat. 2013;62(4):609–24. 25089067 10.1111/fare.12030 PMC4116141

[80] Taylor RJ, Hernandez E, Nicklett EJ, Taylor HO, Chatters LM. Informal Social Support networks of African American, latino, Asian American, and native American older adults. In: Whitfield K, Baker T, editors. Handbook of Minority Aging. Springer Publishing Company; 2013. pp. 417–34. 10.1891/9780826109644.0026.

[81] Taylor RJ, Skipper AD, Ellis J, Chatters LM. Older African American, Black Caribbean, and non-latino white fictive kin relationships. Annu Rev Gerontol Geriatr. 2022;41(1):1–32. PMC9005029 35418718

[82] Gallegos ML, Segrin C. Family connections and the latino Health Paradox: exploring the Mediating role of loneliness in the relationships between the Latina/o Cultural Value of Familism and Health. Health Commun. 2022;37(9):1204–14. 10.1080/10410236.2021.1909244. 33853460 10.1080/10410236.2021.1909244

[83] Ruiz ME. Familismo and filial piety among latino and Asian elders: reevaluating family and social support. Hisp Health Care Int. 2007;5(2):81.

[84] LaValley SA, Gage-Bouchard EA. Life Course Stage and Social Support mobilization for end-of-life caregivers. J Appl Gerontol. 2020;39(8):820–7. 10.1177/0733464818766666. 29644908 10.1177/0733464818766666

[85] Thoits PA. Mechanisms linking social ties and support to physical and Mental Health. J Health Soc Behav. 2011;52(2):145–61. 10.1177/0022146510395592. 21673143 10.1177/0022146510395592

[86] Schwarzer R, Leppin A. Social Support and Health: a theoretical and empirical overview. J Soc Pers Relatsh. 1991;8(1):99–127. 10.1177/0265407591081005.

[87] Magasi S. Social support and social network mobilization in older African American women who have experienced strokes. Disabil Stud Q. 2004;24(4).

[88] Taylor RJ, Chatters LM, Mays VM. Parents, children, siblings, In-Laws, and Non-kin as sources of emergency assistance to Black americans. Fam Relat. 1988;37(3):298–304. 10.2307/584566.

[89] Li S (Alex), Hu W, Guo F, editors. Recent Relocation Patterns Among Older Adults in the United States. *J Am Plann Assoc*. 2022;88(1):15–29. 10.1080/01944363.2021.1902842

[90] Havekes E, Bader M, Krysan M. Realizing racial and ethnic Neighborhood preferences? Exploring the mismatches between what people want, where they search, and where they live. Popul Res Policy Rev. 2016;35(1):101–26. 10.1007/s11113-015-9369-6. 26806990 10.1007/s11113-015-9369-6 PMC4716051

[91] Lewis VA, Emerson MO, Klineberg SL. Who we’ll live with: Neighborhood racial composition preferences of whites, blacks and latinos. Soc Forces. 2011;89(4):1385–407. 10.1093/sf/89.4.1385.

[92] Hargrove TW, García C, Cagney KA. The role of neighborhoods in shaping the aging experience during Times of Crisis. Public Policy Aging Rep. 2021;31(1):38–43. 10.1093/ppar/praa041. 33462555 10.1093/ppar/praa041 PMC7798696

[93] Hayward RA, Morris Z, Otero Ramos Y, Silva Díaz A. Todo ha sido a pulmón: community organizing after disaster in Puerto Rico. J Community Pract. 2019;27(3–4):249–59. 10.1080/10705422.2019.1649776.

[94] Alawiyah T, Bell H, Pyles L, Runnels RC. Spirituality and faith-based interventions: pathways to disaster resilience for African American Hurricane Katrina survivors. J Relig Spiritual Soc Work Soc Thought. 2011;30(3):294–319. 10.1080/15426432.2011.587388.

[95] Garcia-Hallett J, Like T, Torres T, Irazábal C. Latinxs in the Kansas City Metro Area: policing and criminalization in ethnic enclaves. J Plan Educ Res. 2020;40(2):151–68. 10.1177/0739456X19882749.

[96] Islam SJ, Kim JH, Baltrus P, et al. Neighborhood characteristics and ideal cardiovascular health among black adults: results from the Morehouse-Emory Cardiovascular (MECA) Center for Health Equity. Ann Epidemiol. 2022;65:120..e1-120.e10. 10.1016/j.annepidem.2020.11.009 PMC8178422 33285258

[97] Gary-Webb TL, Egnot NS, Nugroho A, Dubowitz T, Troxel WM. Changes in perceptions of neighborhood environment and cardiometabolic outcomes in two predominantly African American neighborhoods. BMC Public Health. 2020;20(1):52. 10.1186/s12889-019-8119-9. 31937271 10.1186/s12889-019-8119-9 PMC6961335

[98] Elo IT, Mykyta L, Margolis R, Culhane JF. Perceptions of Neighborhood Disorder: the role of Individual and Neighborhood Characteristics*. Soc Sci Q. 2009;90(5):1298–320. 10.1111/j.1540-6237.2009.00657.x. 20174462 10.1111/j.1540-6237.2009.00657.x PMC2822409

[99] Kim D. Blues from the Neighborhood? Neighborhood characteristics and Depression. Epidemiol Rev. 2008;30(1):101–17. 10.1093/epirev/mxn009. 18753674 10.1093/epirev/mxn009

[100] Victor CR, Pikhartova J. Lonely places or lonely people? Investigating the relationship between loneliness and place of residence. BMC Public Health. 2020;20(1):778. 10.1186/s12889-020-08703-8. 32456626 10.1186/s12889-020-08703-8 PMC7251825

[101] Kearns A, Whitley E, Tannahill C, Ellaway A, ‘Lonesome, town’?, Is loneliness associated with the residential environment, including housing and neighborhood, factors?. J Community Psychol. 2015;43(7):849–67. 10.1002/jcop.21711. 10.1002/jcop.21711 PMC4699260 26740728

[102] Thomas Tobin CS, Huynh J, Farmer HR, et al. Perceived Neighborhood racial composition and depressive symptoms among Black americans Across Adulthood: evaluating the role of Psychosocial risks and resources. J Aging Health. 2023;35(9):660–76. 10.1177/08982643221100789. 35657773 10.1177/08982643221100789 PMC10478356

[103] Auspurg K, Schneck A, Hinz T. Closed doors everywhere? A meta-analysis of field experiments on ethnic discrimination in rental housing markets. J Ethn Migr Stud. 2019;45(1):95–114. 10.1080/1369183X.2018.1489223.

[104] Pager D, Shepherd H. The sociology of discrimination: racial discrimination in employment, Housing, Credit, and Consumer Markets. Annu Rev Sociol. 2008;34(34, 2008):181–209. 10.1146/annurev.soc.33.040406.131740. 20689680 10.1146/annurev.soc.33.040406.131740 PMC2915460

[105] Crewe SE. Aging and gentrification: the Urban experience. Urban Soc Work. 2017;153–64. 10.1891/2474-8684.1.1.53.

[106] Versey HS. A tale of two harlems: Gentrification, social capital, and implications for aging in place. Soc Sci Med. 2018;214:1–11. 10.1016/j.socscimed.2018.07.024. 30125754 10.1016/j.socscimed.2018.07.024

[107] Pearlin LI, Schieman S, Fazio EM, Meersman SC. Stress, Health, and the Life Course: some conceptual perspectives. J Health Soc Behav. 2005;46(2):205–19. 10.1177/002214650504600206. 16028458 10.1177/002214650504600206

[108] Thoits PA. Stress and health: major findings and policy implications. J Health Soc Behav. 2010;51(1suppl):S41–53. 10.1177/0022146510383499. 20943582 10.1177/0022146510383499

[109] Welch V, Ghogomu ET, Dowling S, et al. In-person interventions to reduce social isolation and loneliness: an evidence and gap map. Campbell Syst Rev. 2024;20(2):e1408. 10.1002/cl2.1408. 10.1002/cl2.1340 PMC10286723 37361556

