# Supplementary material for: Institutional, neighborhood, and life stressors on loneliness among older adults
Source: BMC Public Health. 2025 Jan 29;25:363. doi: 10.1186/s12889-025-21463-7 (PMC11776274; doi:10.1186/s12889-025-21463-7)
Supplement: Supplementary file 1 — Supplementary Material 1 [file 12889_2025_21463_MOESM1_ESM.docx]

| **Supplemental Table 1. Pearson Correlations of Loneliness, Perceived Institutional Discrimination, Stressful Life Events, Neighborhood Discohesion and Disorder, Income, and Education** | | | | | | | |
| --- | --- | --- | --- | --- | --- | --- | --- |
| **Variables** | **1** | **2** | **3** | **4** | **5** | **6** | **7** |
| **1. Loneliness** | 1.00 |  |  |  |  |  |  |
| **2. Perceived Institutional Discrimination Total** | 0.11 | 1.00 |  |  |  |  |  |
| **3. Stressful Life Events Total** | 0.10 | 0.22 | 1.00 |  |  |  |  |
| **4. Neighborhood Discohesion** | 0.27 | 0.14 | 0.17 | 1.00 |  |  |  |
| **5. Neighborhood Disorder** | 0.17 | 0.13 | 0.16 | 0.76 | 1.00 |  |  |
| **6. Income** | -0.14 | -0.03 | -0.10 | -0.16 | -0.18 | 1.00 |  |
| **7. Years of Education** | -0.12 | 0.08 | 0.02 | -0.13 | -0.18 | 0.35 | 1.00 |
| **Note:** All correlation coefficients in the table are statistically significant at the 0.05 level. | | | | | | | |
